# Suicide Prevention based on IA detection for Inpatients (SPIIN)

**DOI:** 10.1192/j.eurpsy.2025.1419

**Published:** 2025-08-26

**Authors:** S. Berrouiguet, C. Apprederisse, L. Carvalho-Fontes, M. Irles

**Affiliations:** 1 Adult Psychiatry, CHU Brest; 2 Human Science department, ISEN, Brest, France

## Abstract

**Introduction:**

Hospital suicide rates vary according to study from 100 to 550 per 100,000 admissions. This is 10 to 30 times the rate of suicide rate in the general population. In prison, there is an average of one death every two or three days, Most of them by suicide. Sensors and Artificial Intelligence to avoid and/or interrupt suicide in the inpatients population. Preliminary explorations will determine weather this kind of device might be acceptable for care providers and patients.

**Objectives:**

Determine weather careproviders (doctors, nurses) consider the SPIN system as usable in a routine hospilat care setting

Determine weather careproviders (doctors, nurses) consider the SPIN system as acceptable

Establish the technical feasability of the SPIN sytem includin hadware (sensors) and softwar (algorithm) in an invitro setting.

**Methods:**

A full presentation of the spin device was presented to the careproviders in a in vitro setting and via a video tutorial of the data capture procédure.

Participants were interviewed during a personal meeting.

Evaluation criteria were : Score to the SUS usablity scale, score the net promoter score and Analysis of the qualitative contents of the interviews.

**Results:**

Qualitative analysis of the interviews contents showed a good

The Spin system will enable earlier intervention in cases where risk factors associated with increased suicidal risk are identified, as well as adjustment of therapies associated with the management of suicidal risk. It can also be used to adjust hospitalization conditions for patients at risk of suicide. transfer knowledge to the prison environment, for example, or to the home of a patient identified as being at high risk (repeat patient).

According to participants, the SPIN device will:

- alleviate the mental workload of caregivers whose job it is to supervise high-risk patients (risk of falling, risk of agitation, risk of suicide, etc.).
- admit patients under better conditions (less frequent searches on admission, less frequent room visits, more human-centred care).

This type of system will free us from the logistical constraints associated with the proximity of surveillance rooms and treatment rooms, or even their availability. In this way, patients in intensive care units (monitoring rooms with a view of the nurses’ station) can benefit from more flexible hospital conditions (accommodation), thanks to a remote monitoring system.

**Image:**

**Figure:**
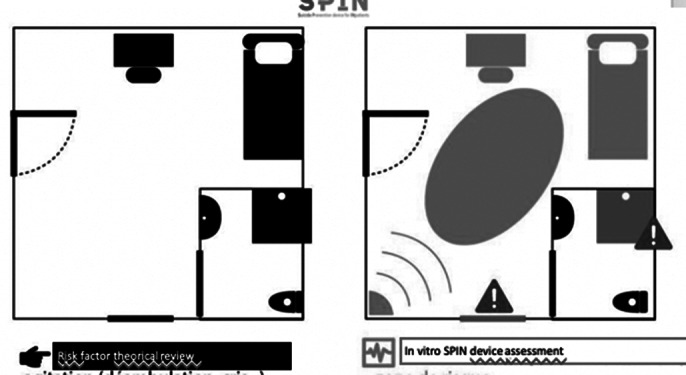

**Image 2:**

**Figure:**
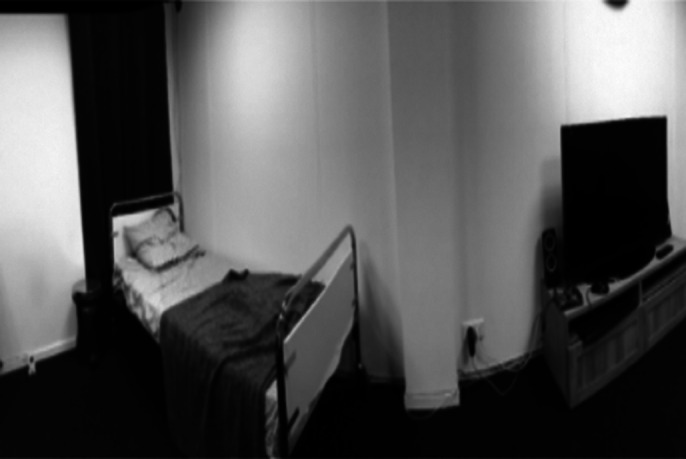

**Conclusions:**

Overall, our study showed a good acceptance of the in vitro setting of the SPIN device in the careproviders population. The newt step of our study will decribe the acceptance in a patient populations and the efficiency on suicidal outcomes in routine population.

**Disclosure of Interest:**

None Declared

